# Le syndrome de la poche à urines violette: à propos de deux cas au Centre Hospitalier Universitaire Régional (CHUR) de Ouahigouya

**DOI:** 10.11604/pamj.2021.38.284.27177

**Published:** 2021-03-18

**Authors:** Siébou Hien, Yérémadé Juste Bonzi, Amidou Sawadogo, Moussa Yanogo, Abibata Rachel Mande, Gérard Coulibaly

**Affiliations:** 1 Service de Médecine Générale, Centre Hospitalier Universitaire Régional de Ouahigouya, Ouahigouya, Burkina Faso,; 2 Service de Néphrologie et d'Hémodialyse, Centre Hospitalier Universitaire Yalgado Ouedraogo, Ouagadougou, Burkina Faso,; 3 Service de Néphrologie et d'Hémodialyse, Centre Hospitalier Universitaire Souro Sanou, Bobo-Dioulasso, Burkina Faso

**Keywords:** Poche à urines violette, urémie, patiente jeune, Ouahigouya, rapport de cas, Purple urine bag, uremia, young patient, Ouahigouya, case report

## Abstract

Phénomène rare survenant le plus souvent chez des patients âgés constipés, porteurs de sonde urinaire chronique et en alitement prolongé, le syndrome de la poche à urine violette traduit généralement une infection du tractus urinaire. Plusieurs bactéries appartenant à deux groupes à la fois (groupe transformant l'indoxyl sulfate urinaire en indoxyl et groupe susceptible d'alcaliniser les urines par la production d'uréase) seraient en cause. Nous décrivons 2 cas au Burkina Faso chez des patientes jeunes de 30 et 16 ans en encéphalopathie urémique avec syndrome infectieux sévère ayant présenté des urines troubles avec une coloration violette de la poche collectrice. Contrairement aux séries rapportées dans la littérature, nos cas sont survenus après les deuxième et quatrième jours de sondage vésical et sur des patientes jeunes sans notion de constipation. Et malgré une antibiothérapie précoce, l´évolution a été défavorable chez une patiente.

## Abstract (en)

Purple urine bag syndrome (PUBS) is an uncommon complication of urinary tract infection. It mainly affects elderly patients with constipation, chronic urinary catheter and prolonged bed rest. Several bacteria belonging to two different groups, the group converting indoxyl urinary sulphate into indoxyl and the group alkalizing urine by the production of urease, are associated with it. We here report two cases of young patients aged 30 and 16 years, living in Burkina Faso, with uremic encephalopathy associated with severe infection and cloudy urine with purple urine bag. Unlike cases reported in the literature, our cases occurred two and four days after urinary catheterization and in young patients without constipation. Despite early antibiotic therapy, outcome was unfavorable in one patient.

## Introduction

Décrit pour la première fois en 1978 par Barlow et Dickson, le syndrome de la poche à urines violette correspond à une coloration soudaine et violette de la sonde et/ou de la poche collectrice d´urines traduisant généralement une infection urinaire sous-jacente [1]. C´est un phénomène rare survenant le plus souvent chez des patients âgés de sexe féminin, en alitement prolongé avec une sonde urinaire et constipation [2-4].

Une étude de revue concernant tous les articles publiés dans PubMed entre octobre 1980 et août 2016 rapporte que l´urémie, l´hypovolémie (choc), le diabète, l´hyperleucocytose et le sexe féminin étaient les facteurs de risque associés à la mortalité chez ces patients [5]. Nous rapportons deux premiers cas au Burkina Faso dans un hôpital universitaire régional chez deux jeunes patientes en insuffisance rénale terminale.

## Patients et observations

**Observation 1:** il s´agit d´une patiente de 30 ans hospitalisée pour altération de l´état général, syndrome urémique avec distension abdominale.

**Antécédents:** patiente déjà hospitalisée du 15 au 27 octobre 2019 dans notre service pour insuffisance rénale terminale (clearance à 1.96ml/min) compliquée d´anémie sévère, troubles phospho-calciques, acidose; mais non prise en charge en dialyse qui n´est pas disponible dans notre contexte.

**Histoire récente de la maladie:** la symptomatologie remonterait au début mars 2020 par une asthénie d´aggravation progressive, anorexie, œdèmes des membres inférieurs, nausées puis vomissements ayant nécessité une hospitalisation dans un service de néphrologie à Ouagadougou où elle aurait reçu un traitement fait de: furosémide injectable, calcium comprimé, bicarbonate de sodium gélule, transfusion de concentré de culot globulaire, antiémétique. Une épuration extra-rénale n´a été effective faute de moyen et de place disponible en dialyse aussi dans ce service. La patiente est donc inscrite dans la liste d´attente en hémodialyse et sortie après stabilisation des signes fonctionnels sus-cités. Trois semaines après son hospitalisation, reprise de la symptomatologie associée à une augmentation du volume de l´abdomen sans notion de fièvre ni de brûlures mictionnelles. Devant l´aggravation des symptômes devenant invalidant, la patiente est conduite aux urgences médicales du CHUR de Ouahigouya le 03 avril 2020 dans un tableau d´encéphalopathie urémique.

**Figure 1:**
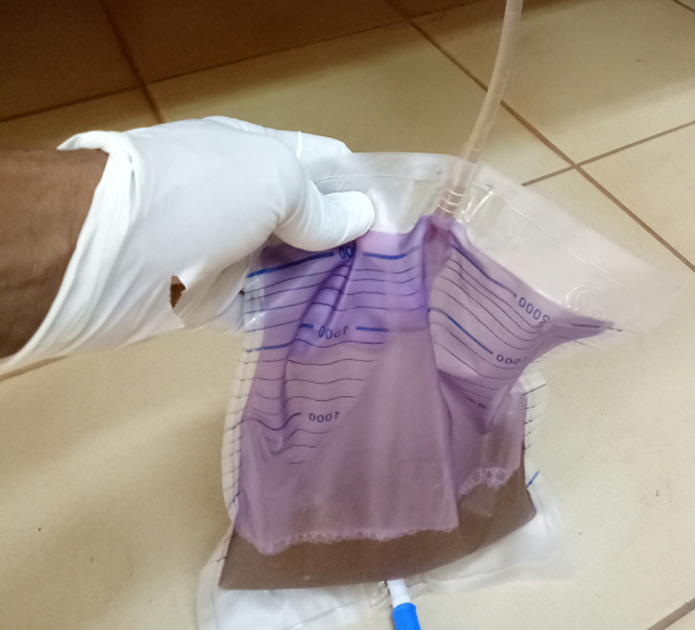
coloration violette de la poche collectrice avec urines troubles

**Evolution et pronostic:** à l´admission aux urgences, l´examen physique note: un mauvais état général avec conscience obnubilée, une léthargie, pression artérielle à 130/96mmHg, une apyrexie, une anémie clinique, un syndrome œdémateux, oligo-anurie. Le bilan d´urgence note une anémie sévère à 5g/dl microcytaire avec leucopénie, une légère hyperkaliémie à 5.8mmol/l, une hypocalcémie à 1.56mmol/l avec hyperphosphatémie à 5mmol/l et acidose avec bicarbonates à 19mmol/l. Elle bénéficie d´une transfusion de concentré de globules rouges (CGR), du furosémide injectable, du calcium injectable, du lévosulpiride injectable, du bicarbonate de sodium gélule, d´une ponction évacuatrice de 1 litre du liquide d´ascite jaune citrin et la mise en place d´une sonde urinaire à demeure. La patiente est ensuite transférée dans le service de médecine générale le 07 avril dans un tableau infectieux avec frissons, odynophagie. L´examen retrouve: une température à 40°C, pression artérielle à 140/90mmHg, distension abdominale, des urines troubles dans la poche collectrice, des amygdales inflammatoires. Devant ce tableau infectieux sévère d´installation brutale un test de diagnostic rapide du paludisme réalisé est revenu positif. Et ne disposant pas de bandelettes urinaires, nous consignons un prélèvement pour examen cytobactériologique des urines (ECBU). Le 08 avril soit 5 jours après la mise de la place de sonde urinaire, nous constatons une coloration violette de la poche collectrice (Figure 1) avec 500ml d´urines toujours troubles.

Vue l´altération de la conscience nous procédons à un changement de la sonde urinaire. Un traitement antipaludique par artésunate injectable ainsi qu´une antibiothérapie probabiliste par amoxicilline acide-clavulanique à raison de 1g toutes les 12 heures furent institués en urgence. Un prélèvement pour étude cytobactériologique des urines note une leucocyturie abondante à 423000 éléments par millilitre et hématurie à 517000 éléments par millilitre mais sans bactériurie, évocateur d´une infection urinaire probablement décapitée. Malgré le traitement antibiotique et antipaludique, l´évolution était marquée par la persistance de l´hyperthermie, des vomissements, de la dysphagie et l´apparition d´un frottement péricardique le 10 avril 2020. Ne disposant pas de traitement d´épuration extra-rénal, seuls des traitements symptomatiques palliatifs étaient effectués. La patiente décède le 13 avril dans un tableau d´encéphalopathie urémique compliquée de choc septique.

**Observation 2:** patiente de 16 ans, hospitalisée pour altération des fonctions rénales dans un contexte d´œdèmes, anémie clinque, crises tonico-cloniques généralisées et poussée hypertensive (180/130mmHg).

**Antécédents:** la patiente ne rapporte aucun antécédent pathologique connu mais rapporte une prise de dermocorticoïdes pour dépigmentation volontaire.

**Histoire de la maladie:** l´histoire remonte au début du mois d´avril 2020 par des œdèmes bilatéraux aux membres inférieurs accompagnés de bouffissure du visage ayant motivé une automédication par des décoctions de feuilles de plantes (nature non précisée) à raison de 1 litre par jour pendant 1 semaine sans amélioration. Elle aurait consulté dans plusieurs formations sanitaires sans succès. Devant l´aggravation des signes associés à des troubles digestifs, une asthénie, des vertiges, une baisse de la diurèse elle fut conduite aux urgences médicales du CHUR de Ouahigouya le 15 juin 2020 où l´examen clinique note: une altération de l´état général, une anémie clinique, un syndrome œdémateux, une élévation de la pression artérielle à 140/90mmHg. Le bilan paraclinique objective une altération des fonctions rénales avec créatininémie à 1959.5μmol/L et urée à 27mmol/L, une anémie sévère normocytaire avec taux d´hémoglobine à 5.5g/dl, une hyperkaliémie à 6.74mmol/L, une acidose avec bicarbonatémie à 14.3mmol/L, une hypocalcémie sévère à 1.1mmol/L avec hyperphosphatémie à 2.44mmol/L. Elle est transférée le 16 juin 2020 au service de médecine générale après transfusion de 250cc de CGR, administration de furosémide injectable, du gluconate de calcium, 500cc de sérum bicarbonaté 14 pour mille, du vogalène injectable.

**Evolution et pronostic:** vingt-quatre heures après son admission dans le service de médecine générale la patiente présente une augmentation de la pression artérielle à 190/140mmHg avec des convulsions tonico-cloniques généralisées faisant évoquer une encéphalopathie mixte (hypertensive et urémique). Ne disposant pas de méthodes d´épuration extra-rénale et devant le manque de moyens financiers pour une évacuation dans un centre plus approprié, nous décidons de procéder à des soins palliatifs par administration de nicardipine injectable puis de l´amlodipine associée à de l´enalapril, du gluconate de calcium injectable relayée par du calcium oral à raison de 2g/24h, du furosémide injectable, du bicarbonate de sodium gélule, une transfusion de 2 poches de 250cc de concentré de globules rouges, du diazépam injectable puis de la lévocétirizine comprimé 5mg par jour, du polystyrène sulfonate de sodium. L´évolution sous ce traitement fut marquée par une amélioration de la pression artérielle mais persistance du syndrome urémique. Au 17^e^ jour d´hospitalisation on note une reprise des convulsions tonico-cloniques avec altération de la conscience.

**Figure 2:**
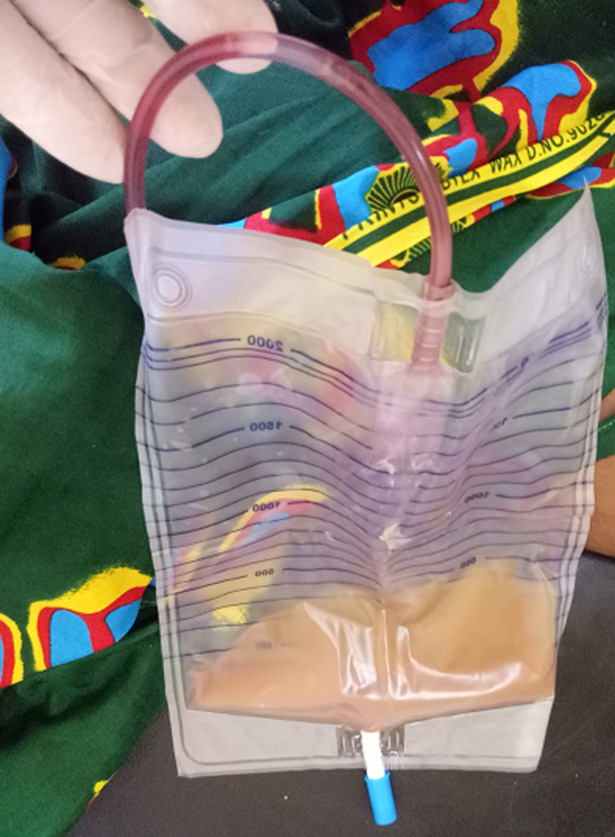
coloration violette de la tubulure et de la poche collectrice contenant des urines purulentes

Ce qui motive la mise en place d´une sonde naso-gastrique d´alimentation et d´une sonde urinaire à demeure. Quarante-huit heures après la pose de la sonde urinaire on note une coloration violette de la poche à urines accompagnée d´une fièvre à 38.6°C et des urines troubles et nauséabondes (Figure 2) avec notion de brûlures mictionnelles. Nous procédons à un changement de la sonde urinaire puis prélèvement pour uroculture et examen à la bandelette urinaire. La bandelette objective: albumine à 3 croix, traces d´hématies, leucocytes à 4 croix, nitrites positives, pH à 8, densité à 1.005, urobilirubine positive, bilirubine à 1 croix et acide ascorbique négatif. L´hémogramme de contrôle note une hyperleucocytose à 37.410 éléments/ml à prédominance neutrophile, une aggravation de l´anémie à 4.8g/dl et une thrombopénie à 93.000 éléments/ml. La protéine C-réactive (CRP) était élevée à 96mg/l. Nous avons débuté une antibiothérapie probabiliste par du ceftriaxone injectable à raison de 1g/12h. L´examen cytologique des urines a retrouvé 720.000 leucocytes/ml, 38.000 hématies/ml, de nombreuses cellules épithéliales, un culot abondant, des cylindres leucoytaires et des cristaux de phosphate. La culture a identifié *Escherichia coli* sensible à la ceftriaxone, au cefuroxime, ciprofloxacine, cefotaxime, cefepime et ceftazidime mais résistant à l´amoxicilline acide clavulanique.

Une semaine après le changement de la sonde urinaire et malgré l´antibiothérapie probabiliste, on note une recoloration de la poche collectrice mais une apyrexie et des urines claires. L´évolution au bout de 2 semaines d´antibiothérapie a été marquée par un amendement clinique et biologique du syndrome infectieux. La pression artérielle était revenue normale sous enalapril 20mg/jour, amlodipine 10mg/jour, furosemide 250mg/jour et clonidine 0.15mg deux fois par jour. Cependant on note une persistance du syndrome urémique (nausées, asthénie, somnolence) avec aggravation de l´altération de la fonction rénale (créatininémie à 3202.6μmol/l). C´est dans ce tableau d´insuffisance rénale terminale d´étiologie indéterminée que la patiente est sortie sous traitement symptomatique avec rendez-vous.

## Discussion

Le syndrome de la poche à urines violette reste un phénomène rare selon les séries dans la littérature [6]. La particularité dans notre série de 2 cas est l´âge jeune des patientes, l´absence de constipation et surtout le délai court (moins d´une semaine) entre la mise en place de la sonde et la survenue de la décoloration. En outre, ces cas surviennent sur un terrain particulier qui est l´insuffisance rénale terminale comme dans celui rapporté par Guei *et al*. [4]. En effet des études ont montré que l´insuffisance rénale chronique est un facteur de risque potentiel dans la survenue du syndrome de la poche à urines violette [7-9]. Bien que la pathogenèse ne soit pas claire, des hypothèses ont été proposées à savoir: l´excrétion du sulfate d'indoxyl principalement dans l'urine, l´augmentation de la concentration sérique et urinaire de ce dernier chez les patients urémiques [7]. Aussi l´infection des voies urinaires chez les hémodialysés ainsi que la pyurie chez les patients oliguriques atteints d’insuffisance rénale chronique (IRC) sont évoqués comme c’est le cas chez nos patientes. *Escherichia coli* isolé chez une de nos patientes fait partie des bactéries les plus fréquemment associées au syndrome [4]. L´une de nos patientes présentait des urines alcalines avec un pH à 8, qui sont également un facteur incriminé.

Bien que considéré comme phénomène bénin dans la plupart des études [2,4,10]. Ce syndrome rarement observé dans notre pratique devrait nous inquiéter surtout lorsqu´il survient sur des terrains particuliers comme les urémiques, les diabétiques, les patients présentant un sepsis avec forte hyperleucocytose ou leucopénie qui sont des facteurs de risque de mortalité [5]. Les facteurs de mortalité retrouvés chez notre patiente étaient l´urémie, le sepsis avec leucopénie.

## Conclusion

Rare, le syndrome de la poche à urines violette peut survenir chez des sujets jeunes sans alitement et cathétérisme urinaire prolongés, ni de constipation. L´urémique est un terrain particulier chez qui nous devrons agir rapidement afin d´éviter une évolution fatale.

## Remerciements

Nous remercions le Laboratoire Privé d'Analyses Biomédicales RAYAN.
